# PPARγ is critical for *Mycobacterium tuberculosis* induction of Mcl-1 and limitation of human macrophage apoptosis

**DOI:** 10.1371/journal.ppat.1007100

**Published:** 2018-06-21

**Authors:** Eusondia Arnett, Ashlee M. Weaver, Kiersten C. Woodyard, Maria J. Montoya, Michael Li, Ky V. Hoang, Andrew Hayhurst, Abul K. Azad, Larry S. Schlesinger

**Affiliations:** 1 Center for Microbial Interface Biology, Department of Microbial Infection and Immunity, The Ohio State University, Columbus, OH, United States of America; 2 Texas Biomedical Research Institute, San Antonio, TX, United States of America; University of Washington, UNITED STATES

## Abstract

Peroxisome proliferator-activated receptor (PPAR)γ is a global transcriptional regulator associated with anti-inflammatory actions. It is highly expressed in alveolar macrophages (AMs), which are unable to clear the intracellular pathogen *Mycobacterium tuberculosis (M*.*tb)*. Although *M*.*tb* infection induces PPARγ in human macrophages, which contributes to *M*.*tb* growth, the mechanisms underlying this are largely unknown. We undertook NanoString gene expression analysis to identify novel PPARγ effectors that condition macrophages to be more susceptible to *M*.*tb* infection. This revealed several genes that are differentially regulated in response to PPARγ silencing during *M*.*tb* infection, including the Bcl-2 family members Bax (pro-apoptotic) and Mcl-1 (pro-survival). Apoptosis is an important defense mechanism that prevents the growth of intracellular microbes, including *M*.*tb*, but is limited by virulent *M*.*tb*. This suggested that *M*.*tb* differentially regulates Mcl-1 and Bax expression through PPARγ to limit apoptosis. In support of this, gene and protein expression analysis revealed that Mcl-1 expression is driven by PPARγ during *M*.*tb* infection in human macrophages. Further, 15-lipoxygenase (15-LOX) is critical for PPARγ activity and Mcl-1 expression. We also determined that PPARγ and 15-LOX regulate macrophage apoptosis during *M*.*tb* infection, and that pre-clinical therapeutics that inhibit Mcl-1 activity significantly limit *M*.*tb* intracellular growth in both human macrophages and an *in vitro* TB granuloma model. In conclusion, identification of the novel PPARγ effector Mcl-1 has determined PPARγ and 15-LOX are critical regulators of apoptosis during *M*.*tb* infection and new potential targets for host-directed therapy for *M*.*tb*.

## Author summary

The bacterium *Mycobacterium tuberculosis* is the causative agent of the disease tuberculosis (TB), which is a global health problem and a leading cause of death world-wide. There is a clear need for better therapies for this disease, the design of which is predicated on better understanding of how *M*. *tuberculosis* interacts with the host. Alveolar macrophages (AMs) are sentinels in the lung which clear inhaled agents, including pollutants, allergens and microbes; yet do not efficiently clear *M*. *tuberculosis*. Here we show that a transcription factor highly expressed in AMs (PPARγ) that is critical for *M*. *tuberculosis* growth inside human macrophages, regulates the anti-cell death protein Mcl-1. We also characterize upstream molecules required for Mcl-1 production and show that PPARγ is important for *M*. *tuberculosis* regulation of cell death. Excitingly, we also show that pre-clinical Mcl-1 inhibitors significantly inhibit *M*. *tuberculosis* growth in human macrophages and multicellular granuloma structures. In summary, here we identify a novel effector of the global regulator PPARγ, determine a role for PPARγ in limiting cell death, and show that the anti-cell death protein Mcl-1 is a promising host-directed target for TB therapy.

## Introduction

Nuclear receptors are a large family of structurally conserved, ligand activated transcription factors, which have a range of functions related to development, homeostasis, metabolism and immunity. Nuclear receptors include receptors for fatty acids such as peroxisome proliferator-activated receptors (PPARs) [1]. PPARs regulate expression of genes involved in fatty acid metabolism and inflammation (pro- and anti-) and are implicated in diabetes, cancer, and infectious diseases, including tuberculosis (TB) [2–6]. Drugs targeting PPARs and other nuclear receptors account for 13% of drugs approved for sale in the US and generated $27 billion in sales in 2009 [7], highlighting their important impact on human health.

There are three PPAR isoforms, α, β/δ, and γ, which are differentially distributed and activated by different ligands. PPARγ is highly expressed in AMs and is important for AM differentiation [8]. PPARγ agonists include oxLDL-derivatives, 15-deoxy-Δ^12,14^-prostaglandin J_2_ (15d-PGJ_2_), 13-hydroxyoctadecadienoic acid (13-HODE), 15-hydroxyeicosatetraenoic acid (15-HETE), and the synthetic thiazolidinediones (TZDs), which are used to treat type 2 diabetes mellitus [9]. PPARs regulate gene expression through multiple mechanisms, including heterodimerizing with the nuclear receptor retinoid X receptor (RXR), and binding to PPAR response elements (PPREs) in the promoter to regulate gene expression [10].

TB is a global threat and leading cause of death worldwide [11]. The increasing incidence of multidrug-resistant (MDR) and extensively drug-resistant (XDR) TB highlights the need for new therapies. There has been an increasing drive towards host-directed therapies (HDTs, [12]), but better understanding of how *M*. *tuberculosis* (*M*.*tb)* interacts with the human host is required for successful design of HDTs [13]. We have demonstrated that *M*.*tb*, and the *M*.*tb* cell wall component mannosylated lipoarabinomannan (ManLAM) enhance PPARγ activity through the mannose receptor (MR) in human macrophages, the host cell niche [4]. PPARγ is critical for *M*.*tb* survival, since knockdown or inhibition of PPARγ reduces *M*.*tb* growth in macrophages [4,5]. Although it is clear that PPARγ contributes to *M*.*tb* survival in human macrophages, the mechanism(s) behind this are incompletely understood.

Apoptosis of infected cells is one important host defense mechanism that prevents the growth of intracellular bacteria and viruses, including *M*.*tb* [14]. Induction of apoptosis is linked to mycobacteria virulence (with less virulent mycobacteria inducing more apoptosis) [15], and induction of macrophage apoptosis leads to reduced *M*.*tb* growth and increased mouse survival [16–18]. However, *M*.*tb* limits apoptosis through mechanisms that are not well understood [19,20]. Host factors that regulate apoptosis include members of the Bcl-2 protein family, which consists of pro-survival (e.g. Bcl-2 and Mcl-1), pro-apoptotic effector (e.g. Bax and Bak), and initiator proteins. Bax and Bak activation is tightly regulated by the initiator and pro-survival proteins, including Mcl-1 [21]. Mcl-1 plays a critical role in regulation of apoptosis and, as such, its activity is tightly regulated at the transcriptional, post-transcriptional, and post-translational levels [22].

Here, we identify new PPARγ effectors through NanoString gene expression analysis, which led to the novel observation of altered expression of genes involved in apoptosis, including the ones encoding Bcl-2 proteins Mcl-1 and Bax. We provide the first evidence that PPARγ regulates expression of Mcl-1 and that this occurs during *M*.*tb* infection and requires 15-lipoxygenase (15-LOX) activity. Further, that PPARγ and 15-LOX are important for *M*.*tb*-mediated limiting of apoptosis. Finally, we determined that pre-clinical therapeutics that target Mcl-1 significantly reduce *M*.*tb* growth in human macrophages and an *in vitro* granuloma model. Thus, herein we identify a novel PAPRγ effector that is a promising HDT target.

## Results

### Identification of PPARγ effectors during *M*.*tb* infection of human macrophages

**Fig 1:**
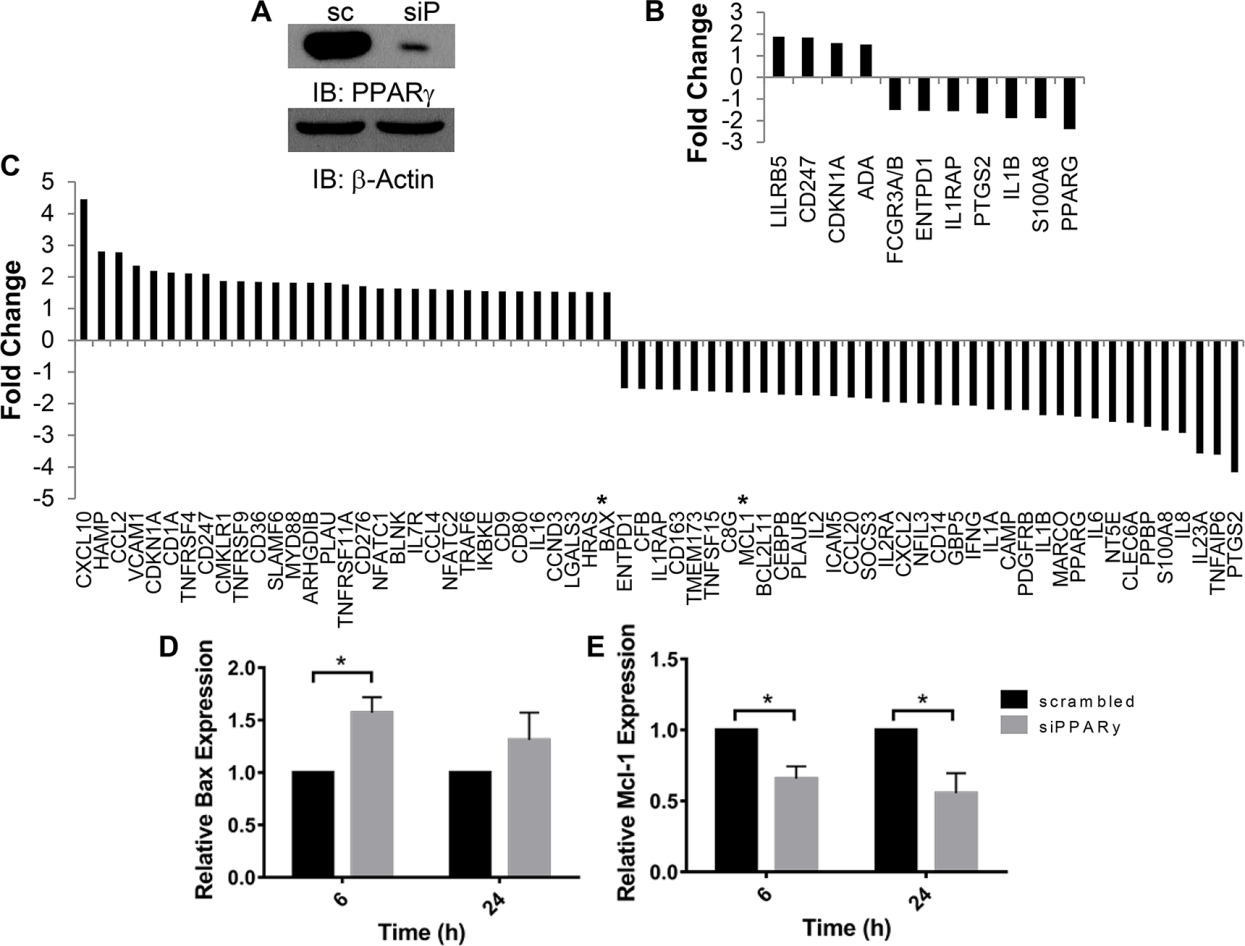
Identification of novel PPARγ effectors with NanoString. MDMs were transfected with PPARγ (siP) or scrambled control (sc) siRNA, then infected with *M*.*tb* at MOI 5 for 6 and 24 h. **A)** Representative Western blot showing knockdown efficiency, mean knockdown efficiency was 81.7 ± 5.5% (N = 3). **B and C)** Total RNA was extracted and NanoString analysis was performed with a Human Immunology Panel. Shown are genes that displayed a significant (*p* < 0.05) mean fold change of at least 1.5 after PPARγ knockdown and 6 **(B)** or 24 **(C)** h of infection. Asterisks in C indicate Bax and Mcl-1. N = 3. **D and E)** Total RNA was collected and gene expression analyzed by qRT-PCR. Results are expressed as Bax **(D)** and Mcl-1 **(E)** expression relative to the scrambled control cells and are the mean ± SEM of 3, in triplicate, * *p* < 0.05.

PPARγ knockdown or inhibition significantly reduces *M*.*tb* growth in macrophages [4,5] through unclear mechanisms. Since PPARγ is associated with anti-inflammatory actions [1,2,9], we hypothesized that PPARγ down-regulates a host protective antimicrobial response to facilitate *M*.*tb* growth. To identify novel PPARγ effectors that facilitate *M*.*tb* growth in macrophages, we undertook expression profiling of genes involved in the immune response using the NanoString nCounter GX Human Immunology Panel. Monocyte-derived macrophages (MDMs) were transfected with scrambled control or PPARγ siRNA (81.7 ± 5.5% PPARγ knockdown, Fig 1A), then infected with *M*.*tb* for 6 and 24 h before cells were lysed and RNA collected. This approach identified multiple immunology-related genes that are significantly changed following PPARγ knockdown and *M*.*tb* infection. At 6 h post infection, 4 genes were significantly increased, and 7 decreased (Fig 1B). More genes were altered at 24 h, with 31 genes significantly increased and 36 genes significantly decreased following PPARγ knockdown (Fig 1C). This included IL-8, which we had previously shown to be regulated by PPARγ during *M*.*tb* infection [4]. Other genes altered following PPARγ knockdown include those involved in IL-1 signaling: IL-1β, IL-1RAP, IL-1α (Fig 1B and 1C). Genes involved in cell death were also differentially expressed following PPARγ knockdown, a conclusion that was corroborated with STRING analysis (S1 Fig). In particular, the pro-apoptotic Bax was increased 1.51-fold and the anti-apoptotic Mcl-1 was reduced 1.65-fold at 24 h.

These results suggested that *M*.*tb* induces Mcl-1 expression and represses Bax expression through PPARγ, to limit apoptosis and enhance *M*.*tb* infection. In support of this, Mcl-1 is induced following *M*.*tb* infection, Mcl-1 is critical for *M*.*tb* limiting of apoptosis and *M*.*tb* survival in human macrophages [17,23], and polymorphisms in the Mcl-1 promoter are linked to TB risk [24]. In contrast, *M*.*tb* infection is correlated with reduced Bax expression [25]. It is unknown if PPARγ regulates Mcl-1 or Bax expression in immune cells. It is also unknown if PPARγ contributes to expression of any of the Bcl-2 family proteins during *M*.*tb* infection. Our NanoString results indicate that *M*.*tb*, which is well established to limit apoptosis [19], may do so through PPARγ-mediated repression of the pro-apoptotic Bax and induction of the anti-apoptotic Mcl-1, novel findings for PPARγ.

### Validation of potential PPARγ effectors in human macrophages

We validated the Bax and Mcl-1 NanoString results with qRT-PCR, and confirmed that PPARγ knockdown led to a significant increase in Bax and decrease in Mcl-1 gene expression during *M*.*tb* infection. This was observed as early as 6 h after *M*.*tb* infection for both Bax and Mcl-1 (Fig 1D and 1E). Besides Mcl-1, *M*.*tb* induces expression of other anti-apoptotic Bcl-2 family members including Bcl-2 and Bcl-xL [26,27]. Since PPARγ induces expression of the anti-apoptotic Bcl-2 in cardiomyocytes and neurons [28,29], we next determined whether PPARγ regulates expression of Bcl-2 or Bcl-xL in human macrophages during *M*.*tb* infection, similarly to Mcl-1. The NanoString results indicated that Bcl-2 expression does not significantly change after PPARγ knockdown (mean 1.03- and 1.26-fold change at 6 and 24 h, respectively). We confirmed this with qRT-PCR, and determined if Bcl-xL is regulated by PPARγ (this gene was not included in the NanoString). Of the three genes, Mcl-1 exhibited the strongest increase with *M*.*tb* infection (S2 Fig). Unlike Mcl-1, Bcl-2 and Bcl-xL expression was not altered following PPARγ knockdown (S2 Fig). These data indicate that PPARγ specifically regulates Mcl-1 expression during *M*.*tb* infection. Mcl-1 plays a critical role in regulation of apoptosis, and due to its short protein half-life (30 min), is tightly regulated at the transcriptional level [22]. In contrast, Bax is highly regulated through post-translational modifications, and exerts redundant activities with Bak [21]. Thus, we chose to focus on PPARγ regulation of Mcl-1.

### PPARγ regulates Mcl-1 expression

**Fig 2:**
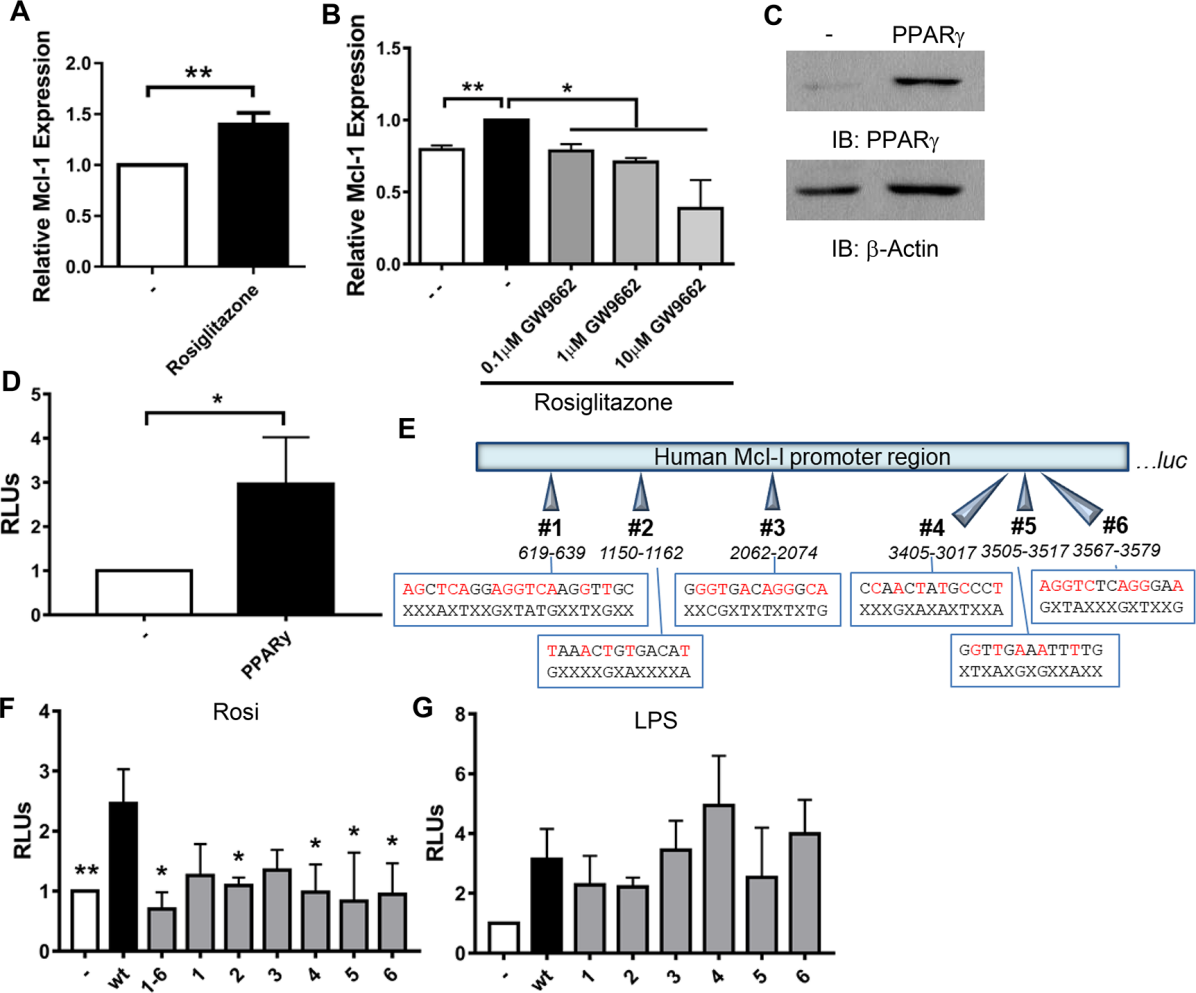
PPARγ induces Mcl-1 expression. **A and B)** MDMs were treated with 100 nM rosiglitazone without **(A)** or with **(B)** 1h pre-treatment with GW9662. After 24 h, total RNA was collected and Mcl-1 gene expression analyzed by qRT-PCR. Results are the mean ± SEM of N = 5 **(A)** or 2 **(B)**. **C, D, F)** RAW cells were transfected with wild-type **(**wt; **C, D, F)** and mutated **(F)** Mcl-1 promoter luciferase reporter constructs, with and without PPARγ expression plasmids. **C)** Western blot was performed to confirm PPARγ expression. **D and F)** After transfection, cells were stimulated with 100 nM rosiglitazone. After 24 h, luciferase activity was determined and normalized to total protein. Results are expressed as (RLUs) normalized to cells not expressing PPARγ and are the mean ± SEM of N = 14 **(D)** or 2–4 **(F)**, * indicate a significant difference between wt reporter and the indicated condition. **E)** Schematic of Mcl-1 promoter region upstream of the luciferase gene (luc) in pGL3. The six putative PPREs are indicated, wt sequences are shown on the top with red indicating consensus to PPRE (AGGTCAnAGGTCA), with mutated sequences below and X indicating no change. **G)** RAW cells were transfected with wt or mutated Mcl-1 promoter luciferase reporter constructs. After transfection, cells were stimulated with 1 μg/ml LPS. After 24 h, luciferase activity was determined and normalized to total protein. Results are expressed as relative luminescence units (RLUs) normalized to cells not stimulated with LPS and are the mean ± SEM of N = 2–3. **A-F)** * *p* < 0.05, ** *p* < 0.01.

We determined if the PPARγ agonist rosiglitazone is sufficient to induce Mcl-1 expression in MDMs and found that rosiglitazone significantly increased Mcl-1 gene expression (Fig 2A). The effect of rosiglitazone was significantly reduced in a dose-dependent manner if MDMs were treated with the PPARγ inhibitor GW9662 (Fig 2B), confirming that rosiglitazone induces Mcl-1 expression through PPARγ and not through off-target effects.

Promoter analysis indicated that the Mcl-1 promoter contains six putative PPREs, indicating that PPARγ may directly regulate Mcl-1 expression. To confirm this, Mcl-1 promoter reporter assays were undertaken in RAW264.7 cells, which express very low levels of PPARγ (Fig 2C; [30]). RAW cells were transfected with a luciferase reporter construct containing the entire Mcl-1 promoter region, with or without PPARγ expression plasmids, then treated with rosiglitazone. Expression of PPARγ resulted in a significant increase in luciferase activity (Fig 2D). To confirm that this is mediated through PPARγ, we mutated the six putative PPREs in the Mcl-1 promoter region (Fig 2E), and repeated the promoter assays. Mutation of any of the six putative PPREs substantially reduced promoter activity, to levels comparable to cells that do not express PPARγ (Fig 2F). This was further reduced if all six PPREs were mutated. In contrast, mutations in any of the six PPREs had no effect on NFκB-driven Mcl-1 expression following LPS treatment (Fig 2G). Together, these data provide evidence that PPARγ enhances expression of Mcl-1, and identifies binding sites for PPARγ in the Mcl-1 promoter.

### *M*.*tb* induces Mcl-1 expression in MDMs and human alveolar macrophages (HAMs)

**Fig 3:**
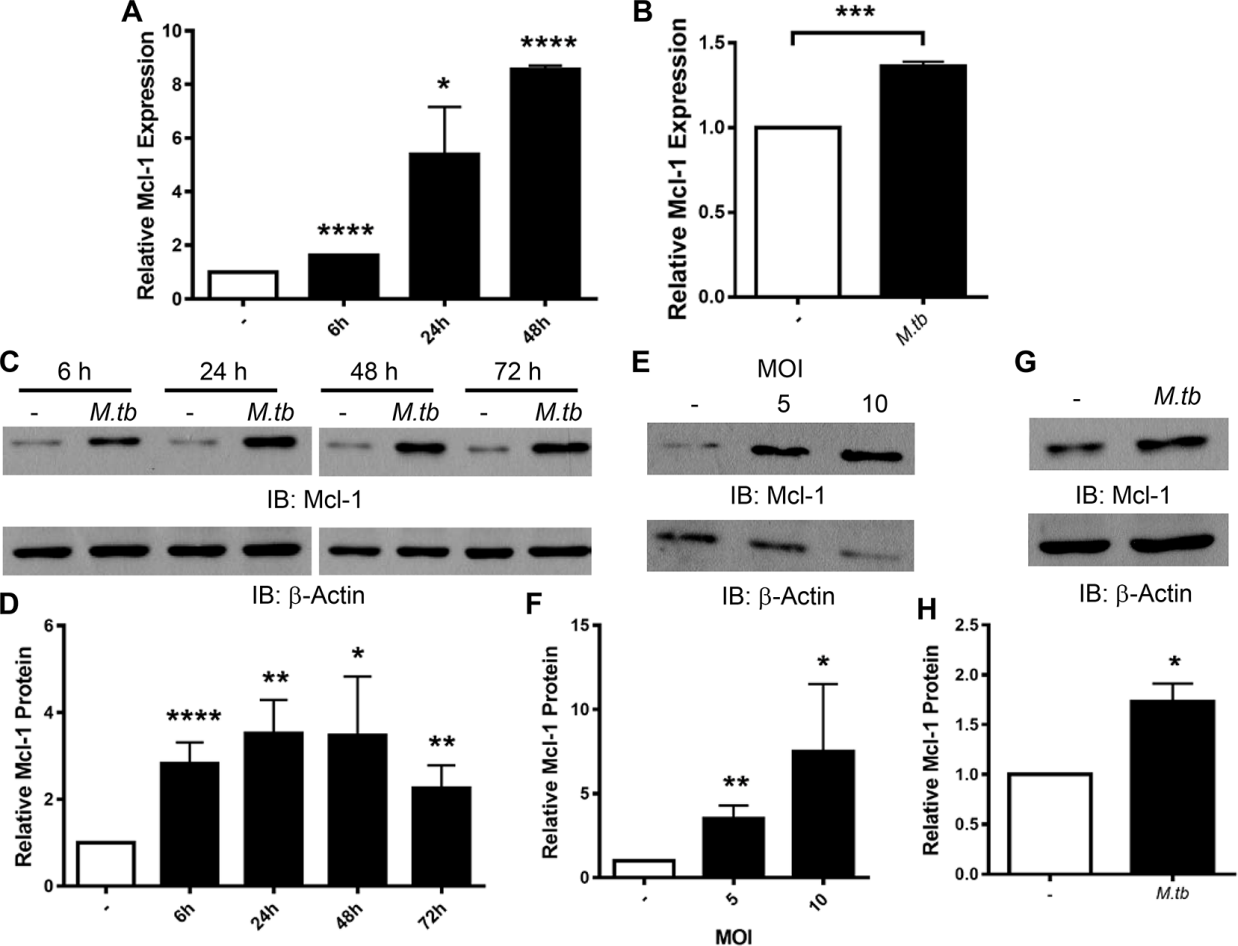
*M*.*tb* induces Mcl-1 expression in human macrophages. MDMs **(A, C-F)** and HAMs **(B, G, H)** were infected with *M*.*tb* at MOI 5 for the indicated times **(A, C, D),** at the indicated MOI for 24 h **(E and F)**, or at MOI 5 for 24 h **(B, G, H)**. **A and B)** Total RNA was collected and gene expression of Mcl-1 analyzed by qRT-PCR. Results are expressed as Mcl-1 expression relative to uninfected cells and are the mean ± SEM of N = 2–3. **C-H)** Protein lysates were collected and analyzed by Western blot **(C, E, G)**. Densitometry analysis of Western blots was conducted with Image J **(D, F, H)**. Data are expressed as amount of Mcl-1 protein relative to uninfected macrophages and are the mean ± SEM of at least 3 experiments. **A-H)** * *p* < 0.05, ** *p* < 0.01,*** *p* < 0.001, **** *p* < 0.0001.

We determined the kinetics of Mcl-1 gene expression in human macrophages during *M*.*tb* infection. We found that *M*.*tb* significantly increased Mcl-1 gene expression in MDMs as early as 6 h post infection, and that this was maintained through at least 48 h (Fig 3A). Similarly, *M*.*tb* infection of HAMs significantly increased Mcl-1 expression (Fig 3B).

We next determined the kinetics of Mcl-1 protein production in human macrophages during *M*.*tb* infection. Similar to gene expression, Mcl-1 protein was induced as early as 6 h after infection with *M*.*tb*, and was maintained for at least 72 h (Fig 3C and 3D). These kinetics are similar to previous reports in human and murine macrophages [17,23,31]. *M*.*tb* at different MOIs (5 or 10) significantly increased Mcl-1 protein 24 h after infection (Fig 3E and 3F). *M*.*tb* also significantly induced Mcl-1 protein in HAMs (Fig 3G and 3H). These data provide the first indication that *M*.*tb* induces Mcl-1 expression in HAMs, which are an important niche for *M*.*tb* during infection.

### PPARγ and 15-LOX are mediators of Mcl-1 production during *M*.*tb* infection

**Fig 4:**
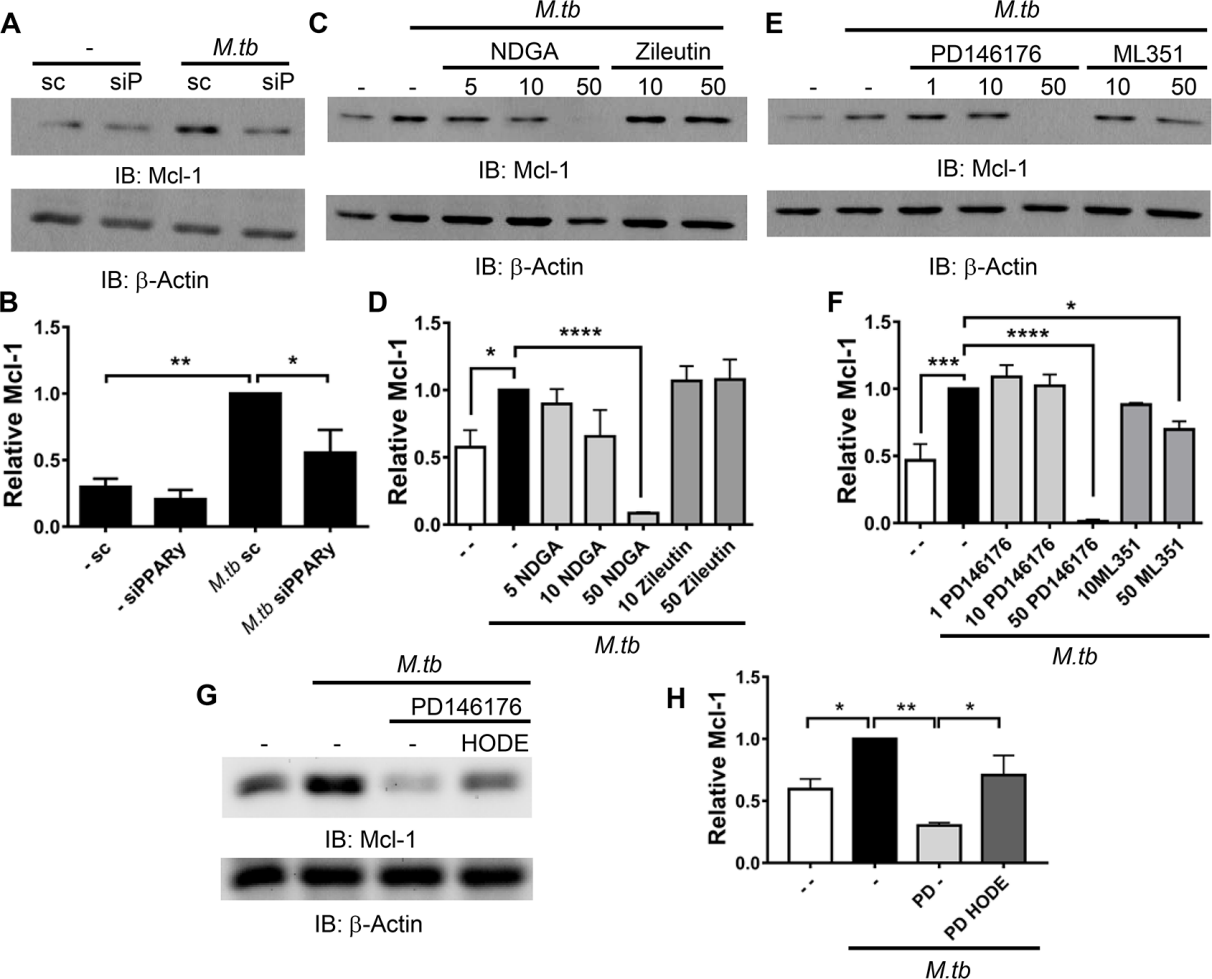
*M*.*tb* induces Mcl-1 in a PPARγ- and 15-LOX-dependent manner. **A and B)** MDMs were transfected with PPARγ (siP) or scrambled control (sc) siRNA then infected with *M*.*tb* at MOI 5 for 24 h. **C-F)** MDMs were treated with the indicated LOX inhibitors (μM) 1 h before, and during, *M*.*tb* infection (MOI 5 for 6 h). **G and H)** MDMs were treated with PD146176 (25 μM) 1 h before, and during, *M*.*tb* infection (MOI 5 for 6 h); along with 13-HODE (30 μM). **A-H)** MDMs were lysed and protein analyzed by Western blot, and densitometry analysis was conducted with Image J. Data are expressed as amount of Mcl-1 relative to *M*.*tb* infected scrambled control **(B)** or infected no inhibitor control **(D, F, H)** and are the cumulative mean ± SEM of at least 3 experiments, * *p* < 0.05, ** *p* < 0.01, *** *p* < 0.001, **** *p* < 0.0001.

We next determined if PPARγ-regulation of Mcl-1 gene expression (Fig 1) would correspond to altered protein levels. MDMs were transfected with PPARγ and scrambled siRNA, then infected with *M*.*tb* for 24 h and protein lysates assessed. As expected, *M*.*tb* induction of Mcl-1 protein production was significantly reduced following PPARγ knockdown (Fig 4A and 4B).

The endogenous ligand for PPARγ during *M*.*tb* infection is unknown. However, previous work by our laboratory indicated that the cytosolic phospholipase A_2_ cPLA_2_ is important for PPARγ activity during *M*.*tb* infection [4]. cPLA_2_ releases arachidonic acid (AA) from the plasma membrane, which can then be converted to the PPARγ agonist 15d-PGJ_2_ through COX-2 or the PPARγ agonists 13-HODE and 15-HETE through 15-lipoxygenase (15-LOX) [9,32], but it is unknown which of these pathways is critical for PPARγ activity in human macrophages. Since *M*.*tb* infection induces COX-2 production [4] and COX-2 has been linked to regulation of Mcl-1 production in human lung adenocarcinoma cells [33], we first determined if COX-2 is important for PPARγ activity and the production of Mcl-1 during *M*.*tb* infection of human macrophages. MDMs were treated with the COX-2 specific inhibitors NS-398 and CAY10404 and then infected with *M*.*tb*. We found that COX-2 inhibition did not reduce Mcl-1 production during *M*.*tb* infection (S3A and S3B Fig). COX-2 is required for PGE_2_ release, and both NS-398 and CAY10404 significantly reduced *M*.*tb*- and LPS-stimulated PGE_2_ release (S3C and S3D Fig), confirming COX-2 inhibition. These results indicate that although *M*.*tb* induces COX-2, this is not important for PPARγ-mediated Mcl-1 production during *M*.*tb* infection of human macrophages and supports the notion that although 15d-PGJ_2_ is a PPARγ agonist, 15d-PGJ_2_ concentrations inside the cell may not reach the levels required to activate PPARγ, a much raised topic in the field [9,34].

Previous work indicated that LOXs are important for PPARγ activation, likely through generation of the PPARγ agonists 13-HODE and 15-HETE [35]. We next determined if LOXs were important for PPARγ activity in human macrophages during *M*.*tb* infection. MDMs were treated with the general LOX inhibitor nordihydroguaiaretic acid (NDGA), and then infected with *M*.*tb*. *M*.*tb*-induced Mcl-1 production was significantly inhibited by NDGA (Fig 4C and 4D). Human macrophages produce two LOX isoforms, 5-LOX and 15-LOX [32,36]. To determine if 5- and/or 15-LOX were critical for PPARγ activity, MDMs were treated with the 5-LOX specific inhibitor zileutin and the 15-LOX specific inhibitors PD146176 and ML351, then infected with *M*.*tb*. 5-LOX inhibition had no effect on Mcl-1 production (Fig 4C and 4D) while Mcl-1 production was significantly reduced following 15-LOX inhibition (Fig 4E and 4F). Together, these data provide evidence that 15-LOX, not 5-LOX or COX-2, is important for PPARγ-mediated Mcl-1 production in human macrophages during *M*.*tb* infection.

To confirm this, we inhibited 15-LOX using PD146176, then stimulated PPARγ by treating cells with the 15-LOX product 13-HODE and probed for Mcl-1 production during *M*.*tb* infection. As expected, we found that 13-HODE ameliorated the reduction of Mcl-1 production seen when 15-LOX is inhibited (Fig 4G and 4H). Taken together, these data provide evidence that 15-LOX, through production of PPARγ ligands, is important for Mcl-1 production in human macrophages during *M*.*tb* infection.

### PPARγ activity limits apoptosis in *M*.*tb*-infected macrophages

**Fig 5:**
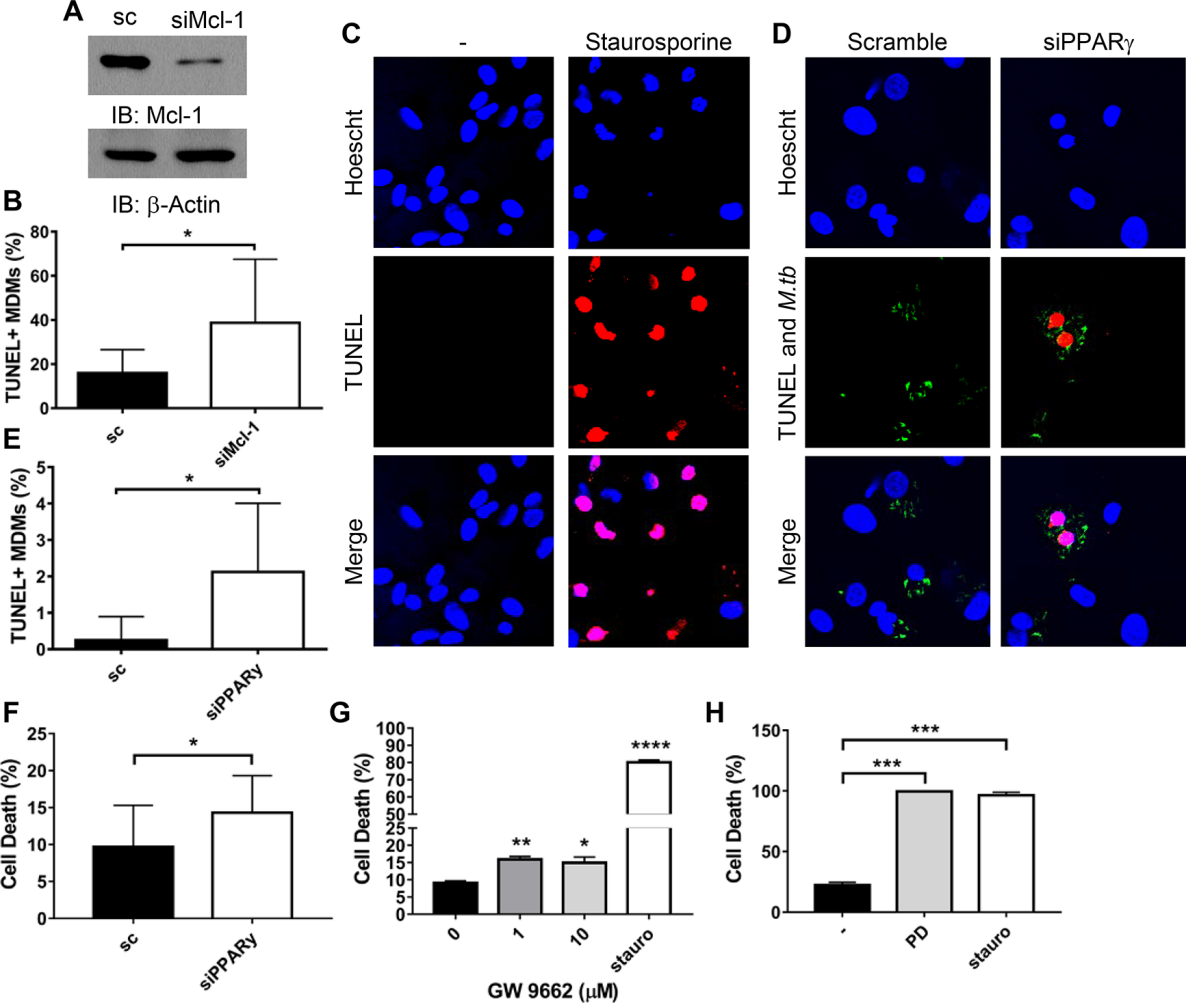
PPARγ and Mcl-1 limit apoptosis during *M*.*tb* infection. **A, B, D, E)** MDMs were transfected with Mcl-1 **(A and B),** PPARγ **(D and E)**, or scrambled control (sc) siRNA then infected with fluorescent *M*.*tb* at MOI 50 for 24 h **(B),** or MOI 5 for 48 h **(D and E)**. Due to the variability amongst donors, these different conditions were necessary to see low levels of apoptosis in the scrambled control cells, mean 8.66 ± 3.69% **(B)** and 2.30 ± 1.69% **(E)**. **A)** Western blot showing Mcl-1 knockdown efficiency, mean knockdown efficiency was 76.6 ± 5.47% (N = 5). **B and E)** Data are representative of 3 experiments and are expressed as percentage of TUNEL^+^ MDMs and are the mean ± SD. The cumulative increase in TUNEL^+^ MDMs following knockdown (N = 3) is shown in S4A and S4B Fig. **C)** MDMs were treated with 5 μM staurosporine overnight then fixed and TUNEL staining performed. **C and D)** Representative images of TUNEL staining, with TUNEL staining indicated in red and fluorescent *M*.*tb* in green. **F, G, H)** MDMs were transfected with PPARγ or scrambled control (sc) siRNA **(F)** or pre-treated with GW9662 **(G)** or PD146176 **(H)** for 1 h, then infected with *M*.*tb* at MOI 5 for 24 h. MDMs were also treated with 5 μM staurosporine for 24 h. Cell death was determined with the CellTiter Glo Assay, data are expressed as % cell death, with uninfected cells set to 0%. Results are mean ± SEM of N = 3 **(F)** or 2 **(H)**, or representative of n = 5 **(G)**. **A-H)** * *p* < 0.05, ** *p* < 0.01, *** *p* < 0.001, **** *p* < 0.0001.

Knockdown of Mcl-1 significantly increases apoptosis during *M*.*tb* infection (Figs 5A, 5B and S4A) [17,23]. Since Mcl-1 is induced by PPARγ (Figs 1, 2, and 4), we hypothesized that PPARγ would also be important for *M*.*tb* limiting of apoptosis. To test this, MDMs were transfected with PPARγ or scrambled control siRNA, then infected with *M*.*tb*, and TUNEL labeling and CellTiter Glo Assays were performed to enumerate apoptotic cells. As a positive control, MDMs were stimulated with the known apoptosis inducer staurosporine [20], which substantially increased MDM apoptosis (Fig 5C, 5G and 5H). Supporting our hypothesis, PPARγ knockdown significantly increased apoptosis during *M*.*tb* infection (Figs 5D, 5E, 5F and S4B). Enumeration of apoptotic cells indicated that > 85% of apoptotic cells were infected with *M*.*tb*. PPARγ inhibition with GW9662 similarly increased cell death during *M*.*tb* infection (Fig 5G). These results indicate that PPARγ, likely through induction of Mcl-1, contributes to *M*.*tb* limiting of apoptosis.

We next determined if 15-LOX, which is critical for PPARγ activity and Mcl-1 expression, contributes to *M*.*tb* limiting of apoptosis. Indeed, we found that the 15-LOX specific inhibitor PD146176 significantly increased cell death during *M*.*tb* infection (Fig 5H). Together, these results indicate that *M*.*tb-*induced Mcl-1 expression, through 15-LOX-dependent PPARγ activity, limits apoptosis in human macrophages.

### Mcl-1 production leads to increased *M*.*tb* growth in macrophages

During *M*.*tb* infection, Mcl-1 knockdown increased apoptosis (Fig 5) [17], which is thought to limit *M*.*tb* infection [19]. Similar to previous reports [4,17,23], we found that Mcl-1 knockdown significantly reduces *M*.*tb* survival in human macrophages (S5A Fig). Thus, we hypothesized that Mcl-1 could serve as a target for HDT to limit *M*.*tb* growth.

**Fig 6:**
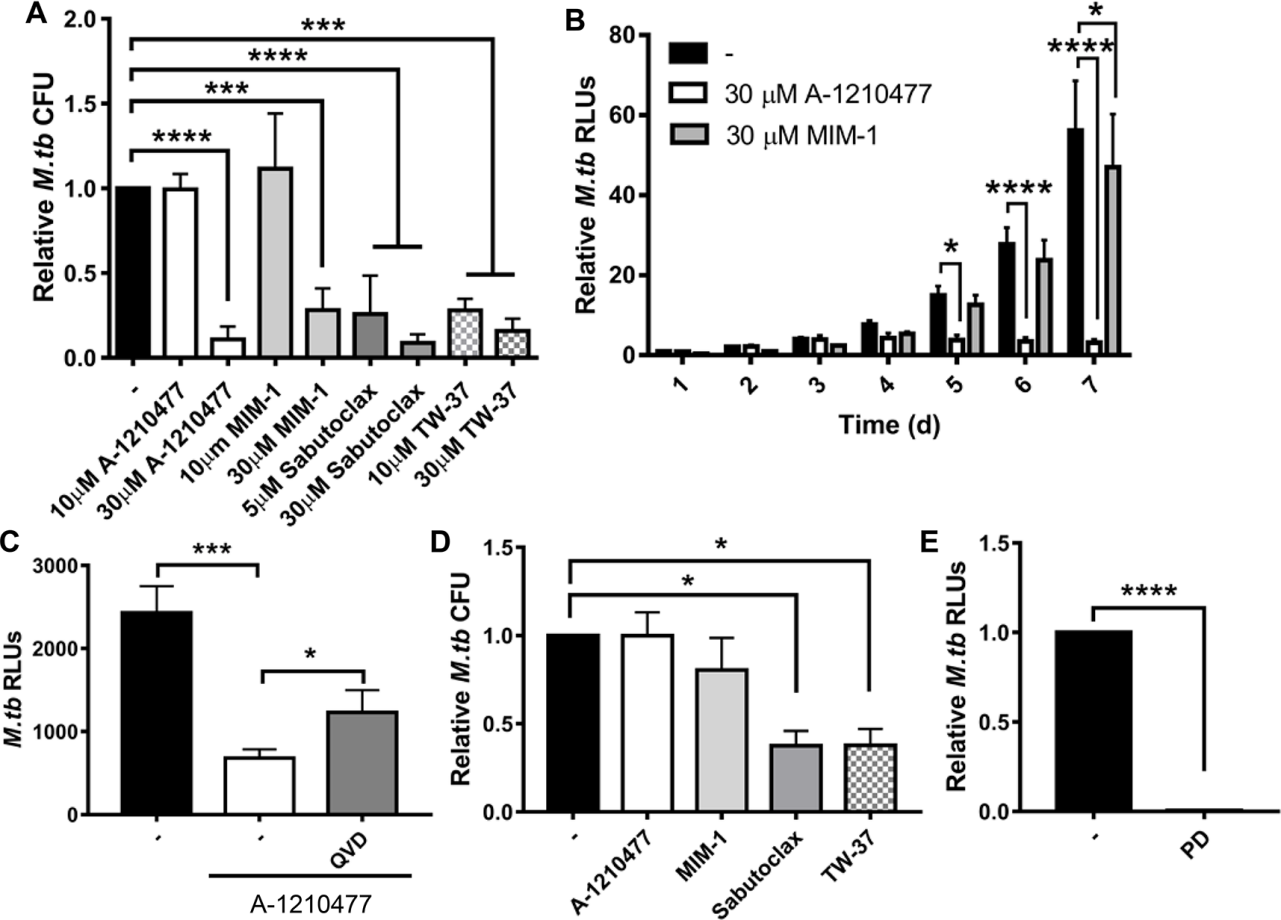
Mcl-1 inhibition limits *M*.*tb* growth. **A)** MDMs were infected with *M*.*tb* at MOI 1, then treated with the indicated Mcl-1 inhibitors. After 4 d, cells were lysed and CFU enumerated. **B)** MDMs were infected with *M*.*tb*-lux at MOI 1, then treated with the indicated Mcl-1 specific inhibitors. *M*.*tb* luciferase activity was measured over time. **C)** MDMs were infected with *M*.*tb*-lux at MOI 1, treated with 100 nM Q-VD-OPH (QVD) for 1 h, then 30 μm A-1210477 and *M*.*tb* luciferase activity was measured after 3 d. Results are the mean ± SD of a representative experiment of 2, in triplicate. **D)** Human PBMCs were infected with *M*.*tb* at MOI 1 to generate *in vitro* TB granulomas, and after 1 day, treated with the indicated Mcl-1 inhibitors (30 μM). After 6 d with inhibitor, cells were lysed and CFU enumerated. **E)** MDMs were infected with *M*.*tb*-lux at MOI 1, then treated with PD146176 (50 μm). After 4 d, *M*.*tb* luciferase activity was measured. **A-E)** Results are the mean ± SEM of N = 3 unless indicated otherwise, * *p* < 0.05, *** *p* < 0.001, **** *p* < 0.0001.

Drugs targeting Mcl-1, and other Bcl-2 pro-survival proteins, are being developed as cancer therapies, and some of these inhibitors have advanced to clinical trials [37]. We queried whether these drugs could be repurposed for TB therapy, by limiting *M*.*tb* growth in macrophages, which has not been studied. MDMs were infected with *M*.*tb*, then treated with the Mcl-1 inhibitors sabutoclax (which targets Mcl-1 and the other pro-survival Bcl-2 proteins), TW-37 (which targets the pro-survival Bcl-2 proteins, and has higher affinity for Mcl-1), A-1210477 and MIM-1 (the latter two are specific for Mcl-1) at concentrations that induce apoptosis [38, 39, 40, 41, 42]. All of the tested Mcl-1 inhibitors significantly reduced *M*.*tb* survival in human macrophages, as determined by CFU enumeration (Fig 6A). The general inhibitors sabutoclax and TW-37 were more potent than the Mcl-1 specific inhibitors, likely due to inhibition of Mcl-1, Bcl-2 and Bcl-xL, which are also induced during *M*.*tb* infection [26,27]. At 30 μM, the Mcl-1 specific inhibitors A-1210477 and MIM-1 reduced *M*.*tb* growth by 89% and 72%, respectively (Fig 6A). Although all of the Mcl-1 inhibitors induce apoptosis, the MDM monolayer remained intact for the time period examined, even at the highest concentration of inhibitor (S5B Fig). We next determined the kinetics of *M*.*tb* growth inhibition for the Mcl-1 specific inhibitors, using a luciferase-expressing *M*.*tb* strain. We noted that by 4 d, both A-1210477 and MIM-1 substantially reduced *M*.*tb* growth with this readout also, and that A-1210477, but not MIM-1, inhibition of *M*.*tb* growth was maintained for at least 7 d (Fig 6B). This is likely due to the binding affinity for Mcl-1, which is in the nM range for A-1210477 and μM range for MIM-1 [37].

We confirmed that the Mcl-1 inhibitors target host responses, since none of the inhibitors reduced *M*.*tb* growth in the absence of macrophages (S5C Fig). To determine if the Mcl-1 inhibitors limited *M*.*tb* growth through apoptosis of host cells, we treated cells with the potent caspase inhibitor Q-VD-OPH to inhibit apoptosis. We observed that caspase inhibition during A-1210477 treatment led to a partial restoration of *M*.*tb* growth (1.52 ± 0.285 fold increase, mean ± SEM, n = 2, Fig 6C), consistent with the notion that Mcl-1 regulates *M*.*tb* growth through apoptosis. These promising results indicate the feasibility of Mcl-1 and other anti-apoptotic Bcl-2 proteins as viable targets for HDT for TB.

A characteristic of TB is formation of granulomas containing macrophages, multinucleated giant cells, lymphocytes, and fibroblasts around *M*.*tb* [13]. These granulomas are thought to help the host contain the bacterium, but also provide a niche for *M*.*tb* that is recalcitrant to antibiotics [13]. We were interested in determining if the Mcl-1 inhibitors could enter the multicellular granuloma structures and limit *M*.*tb* growth in this microenvironment. To assay for this, we used a human *in vitro* granuloma model that we previously characterized, which contains macrophages, multinucleated giant cells, T cells, and B cells [43,44] and provides a unique model system for the study of drugs in a complex human granuloma. We found that the Mcl-1 inhibitors Sabutoclax and TW-37, at concentrations that inhibited growth in human macrophages, significantly reduced *M*.*tb* growth in human *in vitro* granulomas (Figs 6D and S5D), indicating that these drugs can penetrate the multicellular granuloma complex. This was observed at both 3 and 6 days after treatment (Figs 6D and S5D). Similar to the MDMs, the broad-spectrum inhibitors Sabutoclax and TW-37 were most effective in the granulomas, and in contrast to the MDM results, inhibitors A-1210477 and MIM-1 did not significantly reduce *M*.*tb* growth in the granuloma structures. This may indicate that A-1210477 and MIM-1 did not efficiently enter the granuloma complexes (and thus higher concentrations might be required than what we tested) while Sabutoclax and TW-37 did, or that inhibition of the combination of Mcl-1, Bcl-2 and Bcl-xL is required when the bacteria are in multicellular complexes. Either way, these promising results indicate the feasibility of Mcl-1 and other anti-apoptotic Bcl-2 proteins as viable targets for HDT for TB.

Since 15-LOX is important for PPARγ activity and Mcl-1 expression, we wondered if 15-LOX would serve as an additional target for HDT for TB. Interestingly, we found that the 15-LOX specific inhibitor PD146176 significantly limited *M*.*tb* growth in human macrophages (Fig 6E). These results indicate that targeting Mcl-1 expression and activity are both promising options for HDT for TB.

## Discussion

PPARγ is critical for *M*.*tb* intramacrophage growth [4,5], yet the mechanisms behind this are incompletely understood (rev in: [3]). PPARγ is important for lipid body formation, and limiting TNFα and IL-6, and increasing IL-8 and IL-10 secretion during BCG and/or *M*.*tb* infection [4,5,45]. However, it is unclear what other pathways are regulated by PPARγ during *M*.*tb* infection, and their role in regulating *M*.*tb* infection. Gene expression analysis (Fig 1) reveals several new and unexpected potential PPARγ effector proteins in regulating human macrophage responses during *M*.*tb* infection.

**Fig 7:**
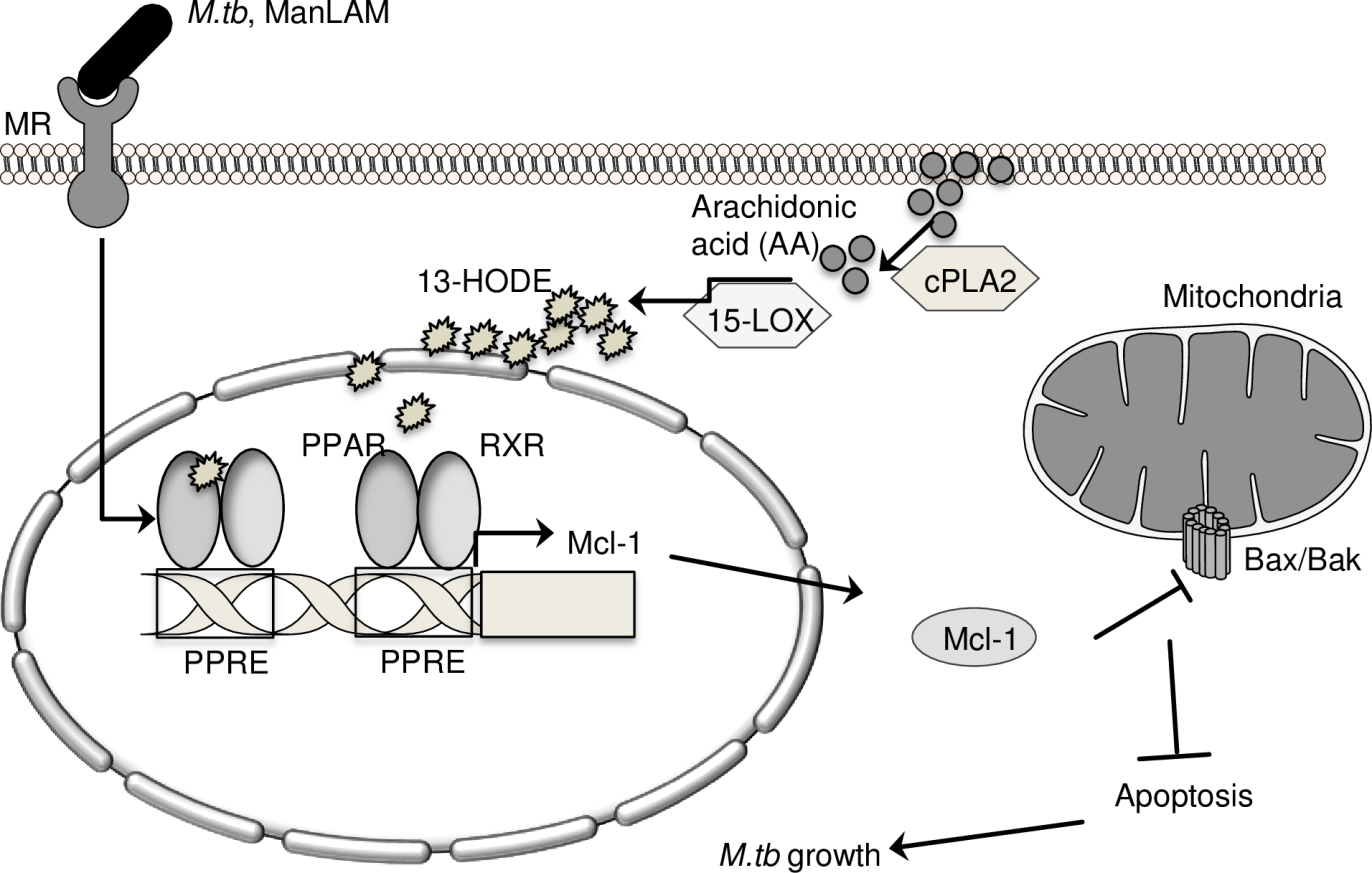
Model. In human macrophages, *M*.*tb* induces Mcl-1 expression through PPARγ, which requires 15-LOX, cPLA_2_, and the mannose receptor (MR) [4]. 15-LOX and PPARγ, through regulation of Mcl-1, contribute to *M*.*tb*’s ability to limit apoptosis and grow in human macrophages.

We show that PPARγ selectively regulates expression of the anti-apoptotic factor Mcl-1. We extend our previous work showing that cPLA2 is important for PPARγ activity [4], by showing that the down-stream enzyme 15-LOX (which contributes to production of the PPARγ agonists 13-HODE and 15-HETE), is important for PPARγ activity and Mcl-1 expression in human macrophages. Mcl-1 limits apoptosis, we show that PPARγ and 15-LOX are also important for *M*.*tb* limiting of apoptosis. Treating macrophages with 15-LOX or Mcl-1 inhibitors significantly reduces *M*.*tb* growth in macrophages and our data showing partial restoration of this phenotype with caspase inhibition is consistent with the notion that Mcl-1 regulates *M*.*tb* growth though apoptosis, although it is possible that other mechanisms also contribute (Fig 7).

Different virulent *M*.*tb* strains (H_37_R_v_, Xinjiang, and the clinical K-strain) and the *M*.*tb* cell wall component ManLAM increase Mcl-1 gene expression [17,27,46,47]. We have previously shown that *M*.*tb* and ManLAM induce PPARγ through the MR [4]. Our current work showing that PPARγ induces Mcl-1 gene expression provides a mechanism for *M*.*tb*- and ManLAM-induced Mcl-1 expression which was previously unknown (activation of MR and PPARγ). The importance of PPARγ in Mcl-1 expression may also explain why heat killed *M*.*tb* H_37_R_v_ does not induce Mcl-1 expression [17], since our lab has previously shown that heat killed *M*.*tb* has significantly reduced ManLAM on the surface and is not recognized by the MR, which is required for PPARγ activation during *M*.*tb* infection [4,48]. Also, the clinical K-strain is more efficient at inducing Mcl-1 expression than *M*.*tb* strain H_37_R_v_ [27]. We have previously shown that ManLAM exposure is highly variable amongst clinical *M*.*tb* isolates [49], suggesting that the *M*.*tb* K-strain has more exposed ManLAM and thus leads to more efficient MR recognition and PPARγ activity, and thus more Mcl-1 induction. In contrast, BCG induces PPARγ less efficiently than *M*.*tb* H_37_R_v_ [4], and does not induce Mcl-1 expression [46,50]. These data all support the notion that *M*.*tb* and ManLAM, through MR and PPARγ, induce Mcl-1 expression, and that mycobacteria that do not efficiently activate PPARγ do not induce Mcl-1 expression. Although PPARγ plays a critical role in regulation of Mcl-1 gene expression during *M*.*tb* infection of human macrophages, *M*.*tb* can regulate Mcl-1 expression through other mechanisms in murine macrophages. For example, in murine macrophages *M*.*tb* activates PKCδ and STAT3, which induce Mcl-1 expression, and *M*.*tb* represses expression of miR-17, which targets Mcl-1 [51]. The Mcl-1 promoter region is highly variable between mouse and man [52] and it is unclear if PKCδ and STAT3 contribute to Mcl-1 induction by *M*.*tb* in human cells. Here, we identified six PPREs in the human Mcl-1 promoter, and the murine Mcl-1 promoter similarly contains putative PPREs, suggesting that although Mcl-1 expression may be regulated differently in murine and human cells, this is potentially driven by PPARγ in both species.

Previous work by our lab indicated that the phospholipase cPLA2 is important for PPARγ activity [4]. cPLA2 mediates release of AA from the plasma membrane, which is utilized to generate various eicosanoids [32]. Altered eicosanoid levels are associated with TB disease progression [53] and enzymes involved in eicosanoid generation contribute to resistance (5-LOX) and susceptibility (COX-2) to *M*.*tb* in animal models [54, 55, 56]. It is largely unknown how these eicosanoids regulate macrophage immune function during *M*.*tb* infection, although some of this is likely through PPARγ since various eicosanoids are suggested to activate PPARγ *in vivo*, including 15d-PGJ_2_ (which requires COX-2) and 13-HODE (which requires 15-LOX) [9,32]. Our current work shows that PPARγ activity and Mcl-1 expression are independent of COX-2 (S3 Fig), but requires 15-LOX (Fig 4), providing evidence that 15-HETE and/or 13-HODE could serve as endogenous PPARγ agonists. This expands our previous work, and highlights that cPLA2 and 15-LOX are both critical for PPARγ activity during *M*.*tb* infection of human macrophages.

Mcl-1 plays a critical role in limiting apoptosis, including during *M*.*tb* infection [17]. Mcl-1 also limits autophagy during *M*.*tb* infection, and reduces *M*.*tb* co-localization with Beclin-1 and lysotracker [31,57]. PPARγ has been linked to induction of autophagy, through increased Beclin-1 expression [58] but the role of PPARγ in regulation of apoptosis is less clear, with studies indicating that PPARγ agonists induce [59–62] and repress [28,29,63,64] apoptosis. This controversy may be due to cell-type specific responses (immortalized cell lines vs primary cells), off-target effects of the PPARγ agonists [59,60] and concentration of agonist used [63]. For example, many studies reporting PPARγ induction of apoptosis use PPARγ agonists at high concentrations, which are known to have off-target effects (or over-express PPARγ, which can also lead to off-target effects), and/or do not confirm that the effect seen with the agonist occurs through PPARγ (using knockdown or inhibition approaches) [61,62]. Indeed, other studies have shown that TZD induction of apoptosis occurs independently of PPARγ, since this occurs in cells that do not express PPARγ or in cells where PPARγ has been inhibited [59,60]. Another study showed high concentrations of PPARγ agonists induce T cell death, while lower concentrations enhance T cell survival; the latter occurred in a PPARγ dependent manner [63]. Here, using PPARγ- and 15-LOX-specific siRNA and/or inhibitors, we show that PPARγ is important for *M*.*tb* limiting of human macrophage apoptosis (Fig 5), and provide a mechanism for this: regulation of Mcl-1 expression.

The pro-survival Bcl-2 proteins, including Bcl-2 and Mcl-1, are highly expressed in various cancers, including follicular lymphoma and chronic lymphocytic leukemia (CLL), and expression levels of multiple pro-survival Bcl-2 proteins, including Bcl-2 and Mcl-1, are correlated with survival outcomes, and resistance to chemotherapeutic agents [65]. As such, targeting these proteins has been a focus of drug development, and clinical trials are underway with promising results for the Bcl-2 specific inhibitor ABT-199—survival of > 2 years for 59% of CLL patients—leading to advancement to Phase III trials [37]. Development and optimization of drugs that target the pro-survival Bcl-2 proteins, including drugs that specifically target Mcl-1 are underway. We capitalized on this active area of research and tested two pan pro-survival Bcl-2 inhibitors that also inhibit Mcl-1 (sabutoclax and TW-37) and two Mcl-1 specific inhibitors (MIM-1 and A-1210477) for their ability to limit *M*.*tb* growth in macrophages and *in vitro* TB granulomas [37]. We show that the Mcl-1 specific inhibitors MIM-1 and A-1210477 significantly limit *M*.*tb* growth in macrophages and that the general Bcl-2 inhibitors sabutoclax and TW-37 also significantly limit *M*.*tb* growth in macrophages and granulomas (Fig 6). Sabutoclax and TW-37 have been successful in cancer xenograft animal models at significantly limiting tumor growth with minimal to no animal toxicity [38,66,67], highlighting the potential use of these inhibitors as therapies for *M*.*tb* infection. Since laboratory and clinical *M*.*tb* strains induce Mcl-1 expression, inhibiting Mcl-1 activity in human cells is expected to limit *M*.*tb* growth, regardless of strain. Targeting these molecules may be a viable HDT option for the various infections that are restricted with apoptosis (e.g. *Streptococcus pneumonia* and *Legionella pneumophila*) [14]. We further show that inhibiting Mcl-1 expression by inhibiting 15-LOX activity significantly reduces *M*.*tb* growth, indicating that targeting Mcl-1 expression and activity represent potential therapeutic routes.

In summary, we have identified a novel PPARγ effector, Mcl-1, and demonstrated upstream mediators of Mcl-1 expression in human macrophages. 15-LOX, PPARγ and Mcl-1 contribute to reducing apoptosis during *M*.*tb* infection, highlighting the importance of the nuclear receptor PPARγ in regulating mediators of apoptosis, and providing a potential mechanism for the critical role of 15-LOX and PPARγ during *M*.*tb* intracellular growth. Importantly, decreased Mcl-1 expression or activity limits *M*.*tb* intracellular growth, opening the door to a new potential HDT target. In this regard, repurposing cancer therapeutics that target Mcl-1 becomes a viable strategy for limiting *M*.*tb* growth in human macrophages and TB granulomas.

## Materials and methods

### Ethics statement

Peripheral blood mononuclear cells (PBMCs) were isolated from human peripheral blood collected from healthy donors, following OSU and Texas Biomed approved IRB protocols. HAMs were isolated from bronchoalveolar lavage of healthy human donors [68], following OSU approved IRB protocols. All donors for these studies provided informed, written consent.

### Isolation and culture of human monocyte-derived macrophages (MDMs) and human alveolar macrophages (HAMs)

MDMs were prepared as described elsewhere [69,70]. Briefly, heparinized blood was layered on a Ficoll-Paque cushion (GE Healthcare, Uppsala, Sweden) to allow for collection of PBMCs. PBMCs were cultured in RPMI (Life Technologies, Carlsbad, CA) with 20% autologous serum in Teflon wells (Savillex, Eden Prairie, MN) for 5 days at 37°C/5% CO_2_. MDMs were harvested and adhered to tissue culture dishes for 2–3 h in RPMI with 10% autologous serum, lymphocytes were washed away, and MDMs were incubated overnight in RPMI with 10% autologous serum. Such MDM monolayers are 99% pure and viable.

### Culture and infection of *in vitro* human granulomas

*In vitro* TB granulomas were generated as described elsewhere [43]. Briefly, human peripheral blood was collected from healthy Mantoux tuberculin skin test (TST) and/or IFNγ release assay (IGRA)-positive individuals. PBMCs were isolated by the published protocol as above, and were immediately infected with *M*.*tb* at MOI 1 in RPMI with 10% autologous serum, then incubated at 37°C/5% CO_2_. After 1 day, Mcl-1 inhibitors were added, and additional serum was added after 4 days. Cells were incubated for a total of 4 or 7 days before *M*.*tb* intracellular growth was enumerated with colony forming units (CFUs), as described below.

### Bacterial strains

Lyophilized *M*.*tb* H_37_R_v_ (27294) was obtained from the American Tissue Culture Collection (ATCC, Manassas, VA). *M*.*tb* H_37_R_v_ lux was created and used as described [71]. This bacterial strain contains the entire bacterial Lux operon cloned in a mycobacterial integrative expression vector. mCherry *M*.*tb* was a kind gift from Dr. Sarah Fortune (Harvard University, Boston, MA). Single cell suspensions of bacteria were prepared as previously described [72,73]. The bacteria concentration and degree of clumping (<10%) were determined with a Petroff-Hausser Chamber. This method results in ≥90% viable bacteria, as determined by CFU assay.

### *M*.*tb* infection of macrophages

Single cell suspensions of *M*.*tb* in RHH [10mM HEPES (Life Technologies) and 0.1% human serum albumin (CSL Behring, King of Prussia, PA) in RPMI] were added to the macrophages at various MOIs and cells were incubated for 2 h at 37°C, with the first 30 min on a platform shaker. Macrophages were then washed and incubated in RPMI with 2% autologous serum for the indicated times. For luciferase-based *M*.*tb* growth assays, MDMs were infected with *M*.*tb*-lux, and bacterial bioluminescence was measured every 24 h for up to 7 days with a GloMax Multi Detection System (Promega, Madison, WI) [71]. Where indicated, MDMs were pre-treated with solvent controls (DMSO), PPARγ (1 h), LOX (1 h), or COX-2 (30 min) inhibitors, with or without the PPARγ agonist 13-HODE (1 h) prior to infection. The PPARγ inhibitor GW9662, the COX-2 inhibitors CAY10404 and NS-398, the 15-LOX inhibitor PD146176, and 13-HODE were purchased from Cayman chemical (Ann Arbor, MI), the 5-LOX inhibitor zileuton was purchased from Sigma (St. Louis, MO), and the LOX inhibitors nordihydroguaiaretic acid (NDGA; pan inhibitor) and ML351 (12, 15-LOX inhibitor) were purchased from Calbiochem (Billerica, MA). The Mcl-1 and caspase inhibitors, or solvent control (DMSO), were added 2 h after infection. For caspase inhibition, cells were pre-treated with 100nM Q-VD-OPH (MP Biomedicals, Santa Ana, CA) 1 h prior to addition of 30 μm A-1210477 and cells were pulsed with 100nM Q-VD-OPH every 24 h. The Mcl-1 inhibitor MIM-1 was purchased from APExBIO (Houston, TX). The Mcl-1 inhibitors A-1210477, TW-37, and sabutoclax were purchased from Selleckchem (Houston, TX). All inhibitors were maintained throughout the course of infection.

### Gene knockdown

MDMs were transfected with 50 nM Accell PPARγ siRNA (GAUUGAAGCUUAUCUAUGA), 50 nM SMARTpool Mcl-1 siRNA (GGUUUGGCAUAUCUAAUAA, GAAGGUGGCAUCAGGAAUG, GAUUAUCUCUCGGUACCUU, CGAAGGAAGUAUCGAAUUU), or the same concentration of scrambled control siRNA (UGGUUUACAUGUCGACUAA, UGGUUUACAUGUUGUGUGA, UGGUUUACAUGUUUUCUGA, UGGUUUACAUGUUUUCCUA) with TransIT-X2 Transfection reagent (Mirus, Madison, WI), following the manufacturer’s recommendations. All siRNAs were purchased from Dharmacon (Lafayette, CO). MDMs were incubated 24 h before use.

### NanoString nCounter mRNA expression profiling

MDMs from three different donors were transfected with scrambled control or PPARγ siRNA and then infected with *M*.*tb* at MOI 5 for 6 and 24 h. MDMs were lysed with TRIzol (Invitrogen, Carlsbad, CA) and total RNA was isolated according to the manufacturer’s recommendations. RNA was analyzed with the NanoString nCounter Human Immunology v2 panel (NanoString, Seattle, WA), which contains primers for 15 housekeeping genes and 579 different immunology-related genes. NanoString processing was performed by the Ohio State University Comprehensive Cancer Center Genomics Shared Resource Core Facility. Data normalization and analysis were performed by the Ohio State University Center for Biostatistics according to the manufacturer’s guidelines using SAS 9.3 and R. Technical normalization was performed using spiked controls, and background was based on the included negative controls. Genes that had counts higher than background were normalized to the housekeeping controls, and fold change in gene expression following PPARγ knockdown was calculated for each donor. Genes that showed a mean fold change of 1.5, with *p* < 0.05 were considered significantly changed. STRING Analysis [74] was performed on genes that were significantly changed after 24 h *M*.*tb* infection.

### Rosiglitazone stimulation of macrophages

Macrophages were stimulated with 100 nM rosiglitazone (Abcam, Cambridge, MA) in RPMI with 2% autologous serum overnight. When indicated, macrophages were pre-treated with 0.1–10 μM PPARγ antagonist GW9662 (Cayman chemical) for 1 h prior to rosiglitazone stimulation. GW9662 was maintained during the rosiglitazone stimulation.

### Western blotting

Cells were washed with PBS, then lysed with TN1 lysis buffer (125 mM NaCl, 50 mM Tris, 10 mM EDTA, 1% Triton X-100, 10 mM Na_4_PO_7_, 10 mM NaF with 10 mM Na_3_VO_4_, 10 μg/ml aprotinin, and 10 μg/ml leupeptin) at 4°C. Lysates were centrifuged (10,000g, 4°C, 10 min) to remove cell debris, then a Pierce BCA assay (Thermo Scientific, Waltham, MA) was performed to determine protein concentration. Equivalent amounts of denatured and reduced protein were separated by SDS-PAGE and analyzed by Western blot using antibodies against PPARγ (C26H12 Cell Signaling, Danvers, MA), Mcl-1 (Santa-Cruz, Dallas, TX), and β-actin (Santa Cruz). Protein band intensities were determined with ImageJ, for each sample background values were subtracted and then values were normalized to the β-actin loading control.

### RNA isolation and gene expression by qRT-PCR

Macrophages in triplicate wells were lysed with TRIzol (Invitrogen) and total RNA was isolated according to the manufacturer’s recommendations. The NanoDrop 1000 was used to determine quantity and quality of RNA. cDNA was reverse transcribed from RNA with SuperScript III Reverse Transcriptase (Invitrogen). Gene expression was determined by quantitative real-time RT-PCR (qRT-PCR) using TaqMan Gene Expression Assays (Applied Biosystems, Foster City, CA) and a CFX96 Real-Time System (Bio-Rad, Hercules, CA). Values were normalized to β-actin, which was used as a housekeeping gene with the Bio-Rad CFX Manager using the ΔΔCq method.

### Mcl-1 promoter assays in RAW264.7 cells

pSV Sport PPARγ2 was a gift from Bruce Spiegelman (Addgene plasmid #8862) [75]. The pGL3-Basic vector containing the full length Mcl-1 promoter region was kindly provided by Dr. Steven W. Edwards ([52]; University of Liverpool, Liverpool, UK) and Dr. Daqing Wu ([76]; Augusta University, Augusta, GA). Six PPRE consensus sequences were identified using Genomatix software [77], and submission to http://www.classicrus.com/PPRE/ and http://www.cbrc.kaust.edu.sa/ppre/. The high complexity of the Mcl-1 region with several repeats, homopolymeric stretches and high GC content demanded a variety of strategies be employed to mutate the various PPRE to non-consensus sequences (summarized in Fig 2E). Sites #4–6 were replaced with gBlocks (IDT, Coralville, IA); site #3 was replaced by SOE-PCR; site #2 was mutated with QuikChange mutagenesis (Agilent Technologies, Santa Clara, CA); site #1 employed limited inverse PCR; all other combinations were subsequently assembled by subcloning. Mutants were fully sequenced through the Mcl-1 region and, where whole plasmid mutagenesis was used, the mutant region was further back cloned to pGL3Mcl-1. RAW 264.7 cells (ATCC TIB-71) were maintained in 10% HI-FBS/0.1% penicillin-streptomycin/DMEM (Life Technologies) and co-transfected with the above PPARγ and Mcl-1 constructs using Lipofectamine 2000 (Life Technologies) according to the manufacturer’s instructions. Cells were stimulated with 0.1 μM rosiglitazone 24 h after transfection, and luciferase activity assayed after an additional 24 h using the Promega Luciferase Assay system. For LPS stimulations, cells were transfected with Mcl-1 constructs as above, then treated with 1 μg/ml LPS for 24 h before luciferase activity was assessed. Protein concentration in the lysates was determined with a Bradford Assay (Bio-Rad) and luciferase activity was normalized to protein concentration for each sample.

### PGE_2_ ELISA

MDMs were incubated with the COX-2 inhibitors for 30 min prior to addition of *M*.*tb* at MOI 5 or 1 μg/ml LPS (Sigma). After 24 h, cell free supernatants were collected and the amount of PGE_2_ in the supernatant was analyzed with a PGE_2_ EIA kit according to the manufacturer’s instructions (Cayman Chemical).

### Apoptosis assays

TUNEL staining: Transfected MDMs on coverslips were infected with mCherry *M*.*tb* at MOI 5 and 50 for 24 and 48 h. Cells were then fixed with 4% PFA (Affymetrix, Santa Clara, CA) and labeled using the Click-iT TUNEL Alexa Fluor Imaging Assay (Invitrogen) following the manufacturer’s instructions. Cells were imaged with an Olympus FV1000 confocal microscope (Olympus, Shinjuku, Japan). Using Olympus Fluoview Viewer, at least 100 MDMs were manually counted to quantify % MDMs that stained with TUNEL.

CellTiter Glo Assays: Transfected or inhibitor treated MDMs in 96 well plates were infected with *M*.*tb* at MOI 5 for 24 h and cell death was assayed in triplicate with the CellTiter Glo Assay (Promega) following the manufacturer’s instructions.

### *M*.*tb* growth assays

Intracellular growth was assayed with two approaches. For CFU assays, infected MDMs were lysed as described previously [78]. Lysates were diluted, and plated on 7H11 agar (BD, Franklin Lakes, NJ). The number of CFUs was enumerated after growth for 3–4 weeks at 37°C. For luciferase growth assays, MDMs were infected with *M*.*tb*-lux, and bacterial bioluminescence was measured in relative luminescence units (RLUs) every 24 h for up to 7 days with a GloMax Multi Detection System (Promega) [71].

For measurement of *M*.*tb* growth in the absence of macrophages, *M*.*tb* was incubated in 7H9 broth (BD) with 30 μM of the Mcl-1 inhibitors. After 4 d at 37°C, *M*.*tb* was diluted and plated on 7H11 agar and CFUs were enumerated after growth for 3–4 weeks at 37°C.

### MDM monolayer integrity

To assess monolayer integrity during the course of experiments, three images per condition were acquired under 40x magnification with phase microscopy (Olympus DP71 microscope digital camera). The total number of cells per field of view was enumerated with ImageJ and then averaged together to calculate relative MDM counts.

### Statistical analysis

Macrophages from at least three different donors were used for each assay, unless indicated otherwise. Although the trend was the same for each donor, the magnitude of change differed among donors. Consequently, results from each experiment were normalized to an internal control and an unpaired one-tailed Student’s *t*-test or ANOVA were performed on the normalized data, with *P* < 0.05 considered significant.

## Supporting information

S1 Fig Genes involved in cell death are differentially expressed following PPARγ knockdown and *M*.*tb* infection. STRING analysis was performed on genes that displayed a significant (*p* <0.05) mean change of at least 1.5 fold after PPARγ knockdown and 24 h *M*.*tb* infection. Genes significantly altered by PPARγ knockdown are shown, with genes involved in cell death in red. The asterisks indicate Bax and Mcl-1.
(TIF)

S2 Fig The anti-apoptotic Bcl-2 and Bcl-xL are not regulated by PPARγ. MDMs were transfected with PPARγ or scrambled control (sc) siRNA, then infected with *M*.*tb*. After 24 h, total RNA was collected and gene expression of Mcl-1 **(A)**, Bcl-2 **(B)**, and Bcl-xL **(C)** analyzed by qRT-PCR. To compare the effect of knockdown during infection, results are expressed as expression relative to scrambled transfected and *M*.*tb* infected cells and are the mean ± SEM of 3–4, in triplicate, *** *p* < 0.001, **** *p* < 0.0001, ns = not significant (*p* > 0.05).
(TIF)

S3 Fig *M*.*tb* induces Mcl-1 in a COX-2-independent manner. **A-C)** MDMs were treated with the indicated COX-2 inhibitors (μM) 30 min before, and during, *M*.*tb* infection (MOI 5 for 24 h). **A, B)** MDMs were lysed and protein analyzed by Western blot, densitometry analysis was conducted with Image J. Data are expressed as amount of Mcl-1 relative to the infected no inhibitor control. **C)** Cell free supernatant was collected and PGE_2_ release enumerated by ELISA. **D)** MDMs were treated with the indicated COX-2 inhibitors (μM) 30 min before, and during, treatment with 1 μg/ml LPS. Cell free supernatant was collected after 24 h and PGE_2_ release enumerated by ELISA. **A-D)** Results are the mean ± SEM of 2 experiments, *** *p* < 0.001, **** *p* < 0.0001.
(TIF)

S4 Fig PPARγ and Mcl-1 limit apoptosis during *M*.*tb* infection. MDMs were transfected with Mcl-1 **(A),** PPARγ **(B)**, or scrambled control (sc) siRNA then infected with *M*.*tb* at MOI 50 for 24 h **(A)** or MOI 5 for 48 h **(B).** Due to different donors, these different conditions were necessary to see low levels of apoptosis in the scrambled control cells. Data are expressed as TUNEL^+^ MDMs relative to scrambled control and are the mean ± SEM of N = 3, * *p* < 0.05.
(TIF)

S5 Fig Mcl-1 is important for *M*.*tb* growth. **A)** MDMs were transfected with Mcl-1 or scrambled control (sc) siRNA then infected with *M*.*tb*-lux at MOI 1. *M*.*tb* luciferase activity was measured over time. **B)** MDMs were infected with *M*.*tb*, then treated with the indicated Mcl-1 inhibitors. After 4 d, images were acquired with a 40x objective, and MDM per field of view was enumerated. **C)** *M*.*tb* was treated with the indicated Mcl-1 inhibitors in 7H9. After 4 d, *M*.*tb* was diluted and CFU enumerated. Results are the mean ± SD of N = 1 of 2 experiments, performed in triplicate; no significant differences were observed. **D)** Human PBMCs were infected with *M*.*tb* at MOI 1 to generate *in vitro* TB granulomas, and after 1 day, treated with the indicated Mcl-1 inhibitors (30 µM). After 3 days with inhibitor, cells were lysed and CFU enumerated. **A-D)** Unless indicated otherwise, results are the mean ± SEM of N = 3, * *p* < 0.05, ** *p* < 0.01, **** *p* < 0.0001, ns = not significant (*p*> 0.05).
(TIF)
