# Supplementary figures and images for: PPARγ is critical for Mycobacterium tuberculosis induction of Mcl-1 and limitation of human macrophage apoptosis
Source: PLoS Pathog. 2018 Jun 21;14(6):e1007100. doi: 10.1371/journal.ppat.1007100 (PMC6013021; doi:10.1371/journal.ppat.1007100)

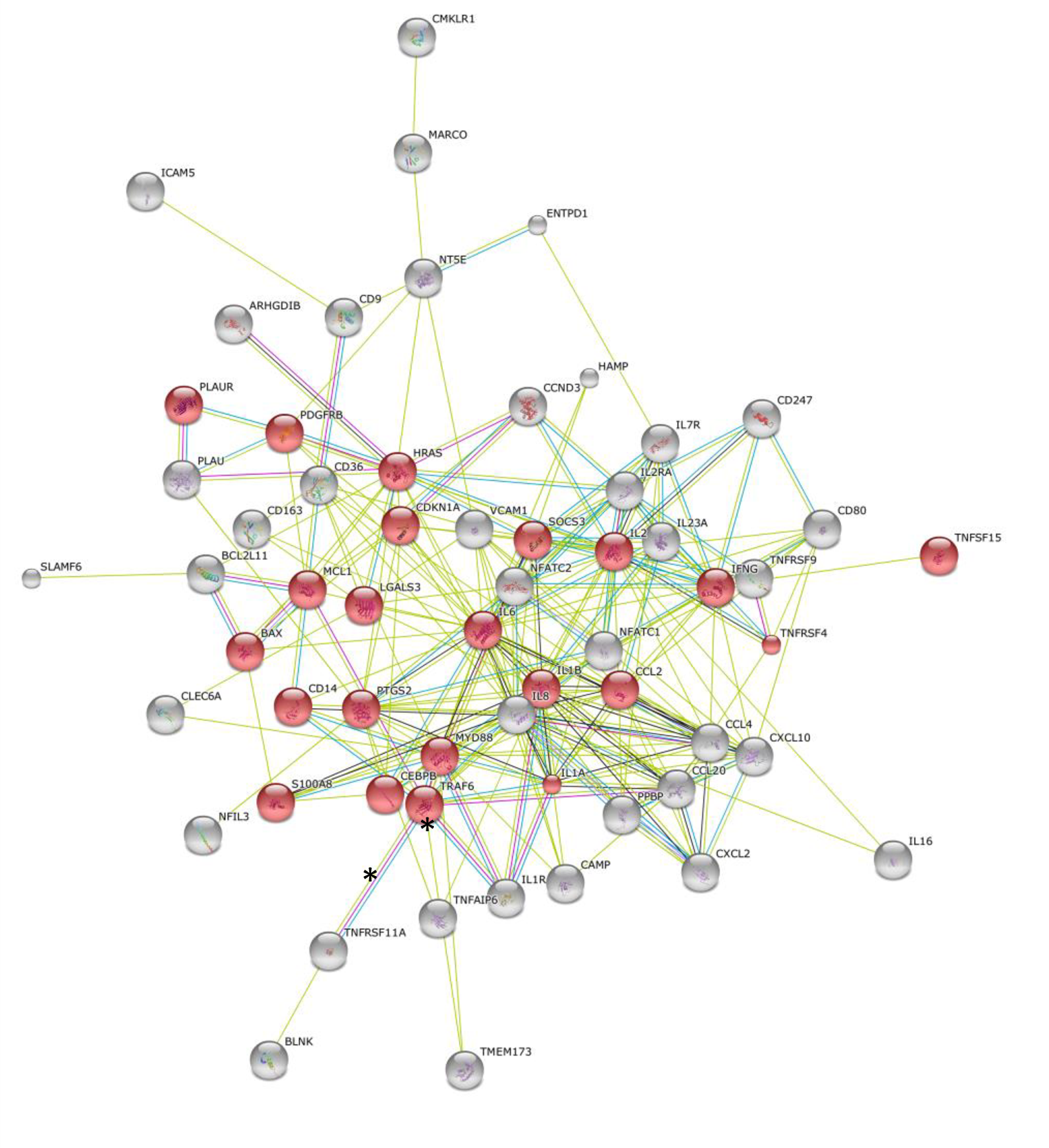

Supplement: S1 Fig — STRING analysis was performed on genes that displayed a significant (p <0.05) mean change of at least 1.5 fold after PPARγ knockdown and 24 h M.tb infection. Genes significantly altered by PPARγ knockdown are shown, with genes involved in cell death in red. The asterisks indicate Bax and Mcl-1. (TIF) [file ppat.1007100.s001.tif]

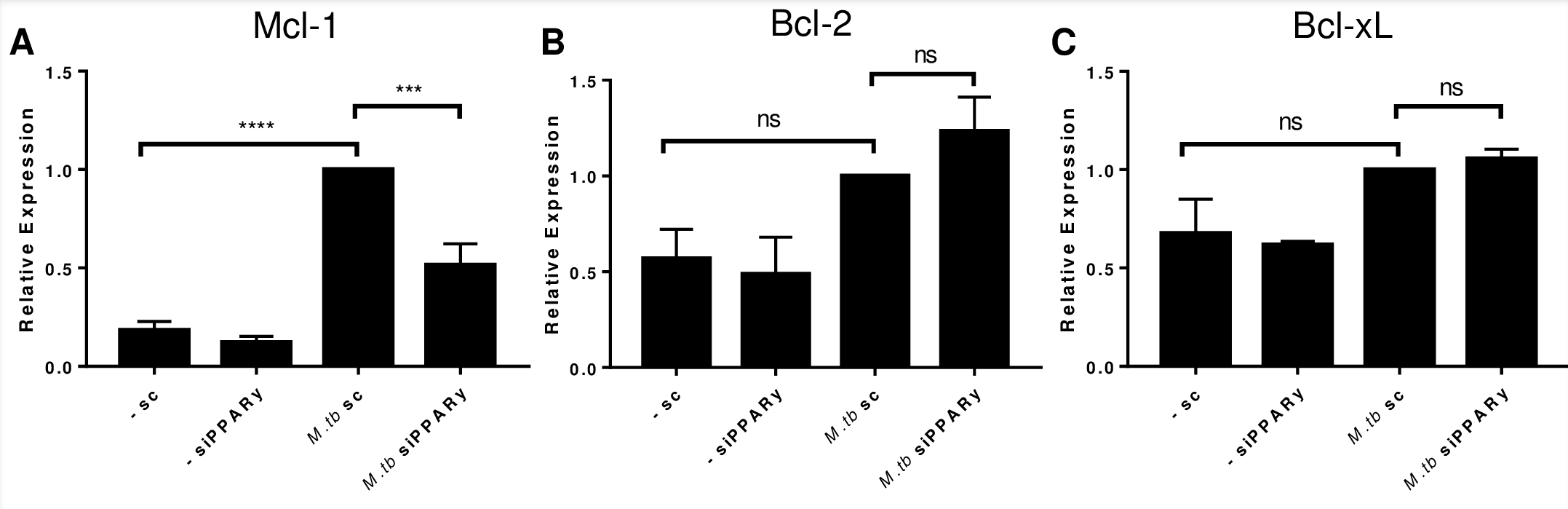

Supplement: S2 Fig — MDMs were transfected with PPARγ or scrambled control (sc) siRNA, then infected with M.tb. After 24 h, total RNA was collected and gene expression of Mcl-1 (A), Bcl-2 (B), and Bcl-xL (C) analyzed by qRT-PCR. To compare the effect of knockdown during infection, results are expressed as expression relative to scrambled transfected and M.tb infected cells and are the mean ± SEM of 3–4, in triplicate, *** p < 0.001, **** p < 0.0001, ns = not significant (p > 0.05). (TIF) [file ppat.1007100.s002.tif]

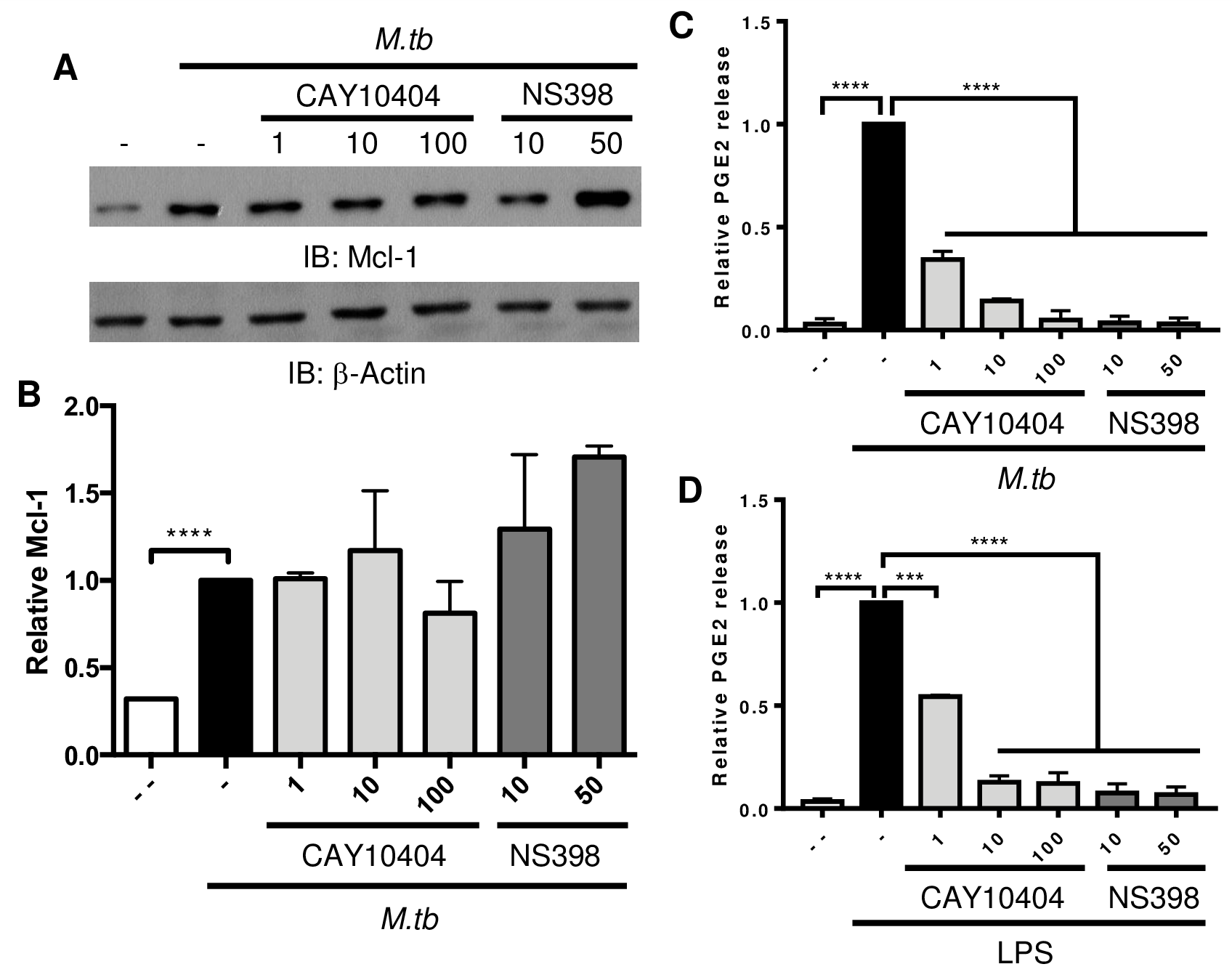

Supplement: S3 Fig — A-C) MDMs were treated with the indicated COX-2 inhibitors (μM) 30 min before, and during, M.tb infection (MOI 5 for 24 h). A, B) MDMs were lysed and protein analyzed by Western blot, densitometry analysis was conducted with Image J. Data are expressed as amount of Mcl-1 relative to the infected no inhibitor control. C) Cell free supernatant was collected and PGE2 release enumerated by ELISA. D) MDMs were treated with the indicated COX-2 inhibitors (μM) 30 min before, and during, treatment with 1 μg/ml LPS. Cell free supernatant was collected after 24 h and PGE2 release enumerated by ELISA. A-D) Results are the mean ± SEM of 2 experiments, *** p < 0.001, **** p < 0.0001. (TIF) [file ppat.1007100.s003.tif]

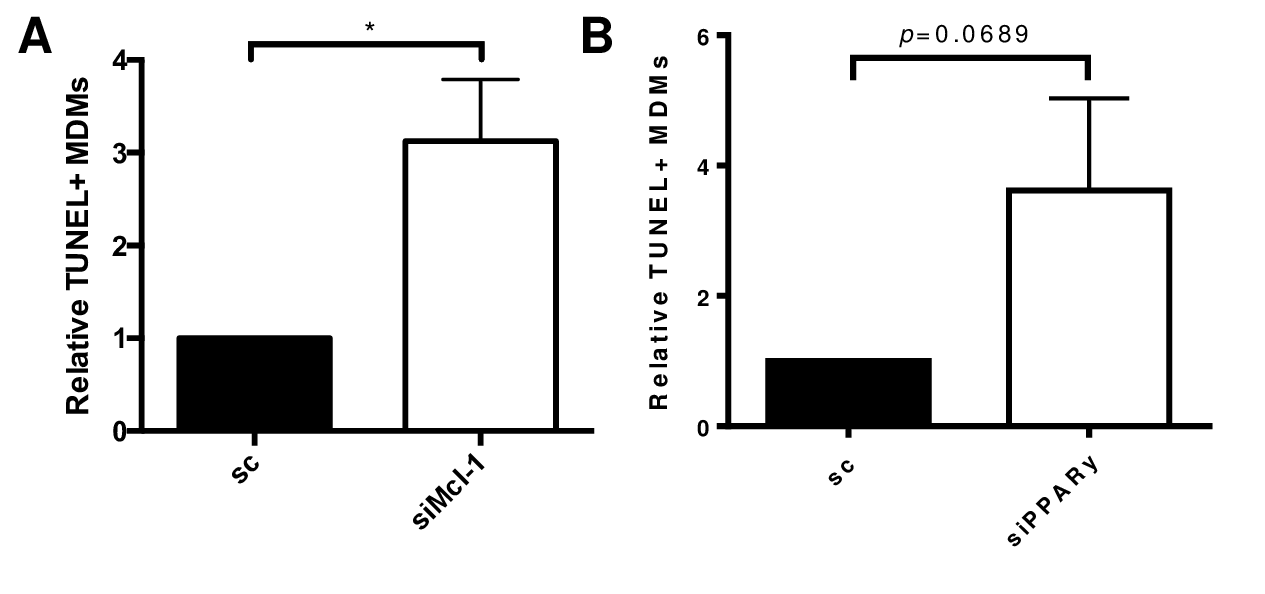

Supplement: S4 Fig — MDMs were transfected with Mcl-1 (A), PPARγ (B), or scrambled control (sc) siRNA then infected with M.tb at MOI 50 for 24 h (A) or MOI 5 for 48 h (B). Due to different donors, these different conditions were necessary to see low levels of apoptosis in the scrambled control cells. Data are expressed as TUNEL+ MDMs relative to scrambled control and are the mean ± SEM of N = 3, * p < 0.05. (TIF) [file ppat.1007100.s004.tif]

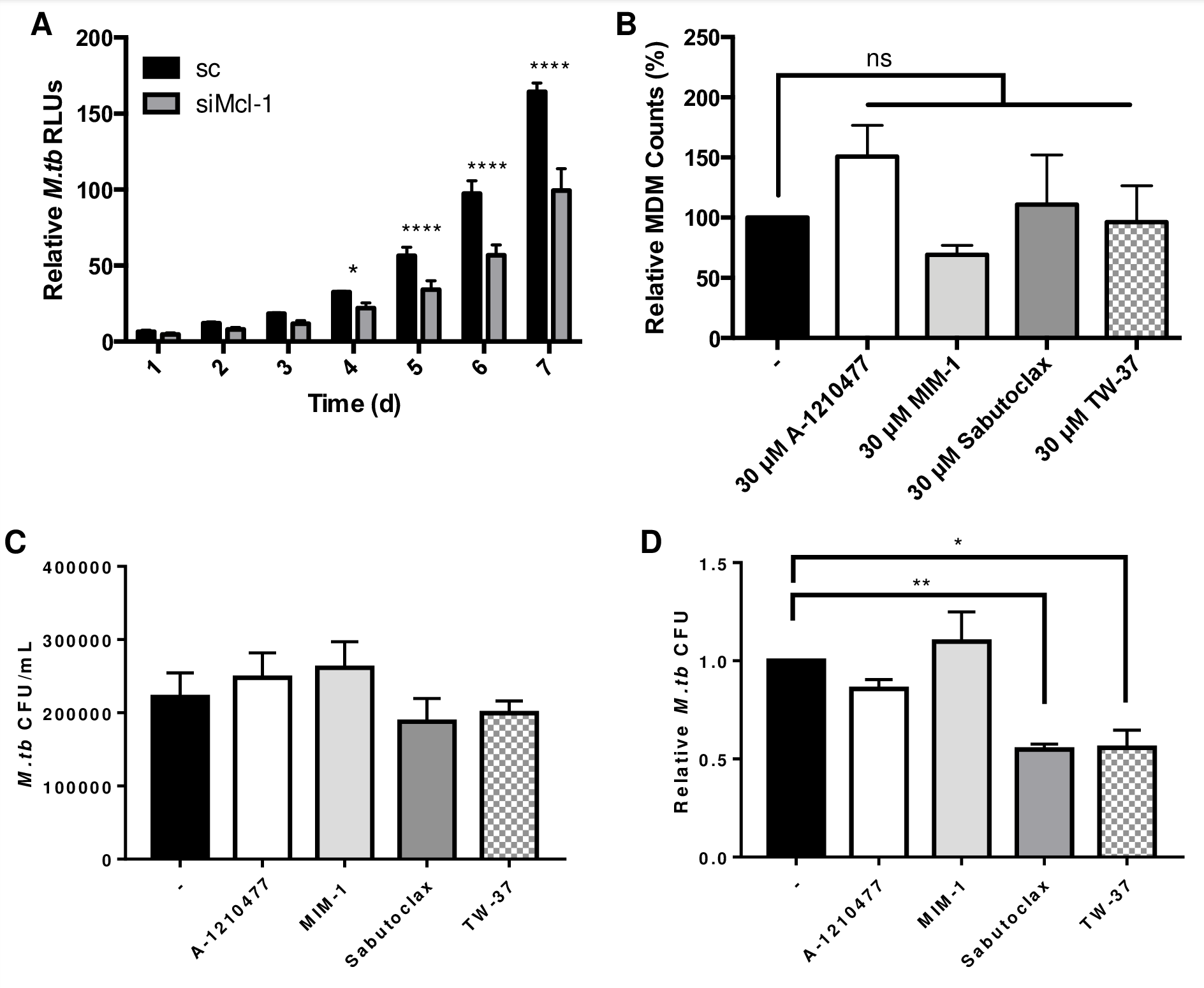

Supplement: S5 Fig — A) MDMs were transfected with Mcl-1 or scrambled control (sc) siRNA then infected with M.tb-lux at MOI 1. M.tb luciferase activity was measured over time. B) MDMs were infected with M.tb, then treated with the indicated Mcl-1 inhibitors. After 4 d, images were acquired with a 40x objective, and MDM per field of view was enumerated. C) M.tb was treated with the indicated Mcl-1 inhibitors in 7H9. After 4 d, M.tb was diluted and CFU enumerated. Results are the mean ± SD of N = 1 of 2 experiments, performed in triplicate; no significant differences were observed. D) Human PBMCs were infected with M.tb at MOI 1 to generate in vitro TB granulomas, and after 1 day, treated with the indicated Mcl-1 inhibitors (30 µM). After 3 days with inhibitor, cells were lysed and CFU enumerated. A-D) Unless indicated otherwise, results are the mean ± SEM of N = 3, * p < 0.05, ** p < 0.01, **** p < 0.0001, ns = not significant (p> 0.05). (TIF) [file ppat.1007100.s005.tif]
